# Over 30 million psychedelic users in the United States

**DOI:** 10.12688/f1000research.2-98.v1

**Published:** 2013-03-28

**Authors:** Teri S Krebs, Pål-Ørjan Johansen

**Affiliations:** 1 Department of Neuroscience, Faculty of Medicine, Norwegian University of Science and Technology, Trondheim, Norway

## Abstract

We estimated lifetime prevalence of psychedelic use (lysergic acid diethylamide (LSD), psilocybin (magic mushrooms), mescaline, and peyote) by age category using data from a 2010 US population survey of 57,873 individuals aged 12 years and older. There were approximately 32 million lifetime psychedelic users in the US in 2010; including 17% of people aged 21 to 64 years (22% of males and 12% of females). Rate of lifetime psychedelic use was greatest among people aged 30 to 34 (total 20%, including 26% of males and 15% of females).

## Introduction

The classical serotonergic psychedelics, LSD (lysergic acid diethylamide), psilocybin (“magic mushrooms”), and mescaline (peyote and other cacti), have their main mechanism of action at the serotonin 2A receptor (5-HT _2A_), produce similar, often indistinguishable subjective effects, and elicit cross-tolerance ^1, 2^. The mechanisms of action, subjective effects, and risk profile of the classical serotonergic psychedelics distinguishes them from other drugs sometimes also labeled “hallucinogens”, such as entactogens, like methylenedioxymethamphetamine (MDMA; ecstasy) that act primarily at serotonin transporters, or dissociative anesthetics, like phencyclidine (PCP) or ketamine that act primarily at NMDA glutamate receptors ^1^.

Prevalence data on psychedelic use in the US is often reported for LSD alone (ignoring psilocybin and mescaline), or for psychedelics grouped together with PCP (popular in the 1970s), MDMA (popular since the 1990s), and/or other “hallucinogens” (some older estimates of hallucinogen use even included cannabis, amphetamine, and cocaine as hallucinogenic drugs), or for use among teenagers but not adults. Here, we present estimated lifetime prevalence of psychedelic use by age category using data from a large US population survey.

## Methods

We examined the estimated lifetime use of psychedelics by age based on 2010 data of the National Survey on Drug Use and Health (NSDUH). Results are presented for males and females separately. We counted participants as having any lifetime psychedelic use if they reported ever using LSD, psilocybin, mescaline, or peyote. The use of mescaline and peyote were combined into one category because mescaline is the active substance of the peyote cactus, but peyote use was also examined separately. Current age was only available as a categorical variable. This study was exempt from review by our Regional Committee for Medical Research Ethics because all data are available in the public domain without any identification of personal information.

### Data source

The annual NSDUH survey provides estimates of substance use and mental health indicators from a randomly-selected sample representative of the US civilian non-institutionalized population aged 12 and older. The Substance Abuse and Mental Health Services Administration (SAMHSA) of the US Department of Health and Human Services is responsible for the NSDUH study design and methods of assessment. Trained interviewers met the randomly-selected participants in their homes, and participants listened to recorded questions via headphones and then entered their answers directly into a computer, providing a highly confidential and standardized setting. The response rate was approximately 78%. In addition, approximately 10% of participants were excluded from the public use data file, either because of excessive missing data on drug use or because they were excluded at random in order to increase anonymity. The total number of respondents in the public use file was 57,873. Detailed information on the sampling and data collection methods, including interview instructions and questionnaires, confidentiality and informed consent are available on the SAMHSA website ( http://oas.samhsa.gov/nsduh.htm).

### Data analysis

Estimates were calculated using the online Survey Documentation Analysis from the Inter-university Consortium for Political and Social Research ( http://dx.doi.org/10.3886/ICPSR32722.v3). Calculations of estimated population percentages and extrapolated total numbers of psychedelic users in the US took into account the weights provided with the NSDUH public use data file. Variance estimates took into account the complex sample design of the NSDUH survey using Taylor series linearization. Respondents with missing data on psychedelic use (less than 1% of the respondents) were assumed to have no use.

## Results

An estimated 32 million (95% confidence interval (CI): 30 to 33 million) US residents in 2010 reported lifetime use of LSD (23 million, 95% CI: 22 to 25 million), psilocybin (21 million, 95% CI: 20 to 22 million), mescaline (11 million, 95% CI: 10 to 12 million), or peyote (6 million, 95% CI: 5 to 7 million).

**Figure 1.**
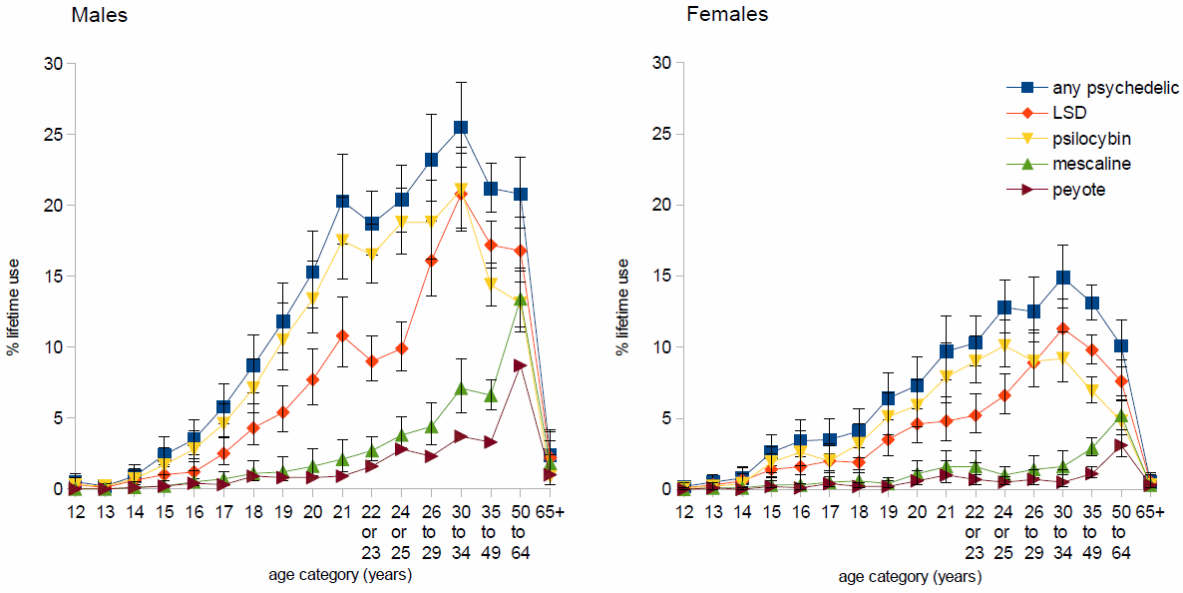
Lifetime psychedelic use by age and gender in the US in 2010. Error bars show 95% confidence intervals. Any psychedelic includes LSD, psilocybin, mescaline, and/or peyote. Mescaline includes both mescaline and peyote.

Figure 1 shows the rate of lifetime psychedelic use in the US in 2010 by age category and gender. Lifetime rate of psychedelic use among people aged 50 to 64 years (the “baby boomer” generation) was similar to the rate among people aged 21 to 49 years. Among people aged 21 to 64 years, 17%, (95% CI: 15% to 18%) reported ever using LSD, psilocybin, or mescaline, including 22% (95% CI: 21% to 23%) of males and 12% (11% to 13%) of females. Prevalence of psychedelic use was low among people aged 65 and older (total 1.3%, 95% CI: 0.8% to 2.1%). Rate of lifetime psychedelic use was greatest among people aged 30 to 34 years (total 20%, 95% CI: 18% to 22%), with 26% (95% CI: 23% to 29%) of males and 15% (95% CI: 13% to 17%) of females.

## Discussion

Psychedelics continue to be widely used in the US. Common reasons given for using psychedelics include curiosity, mystical experiences, and introspection ^3^. Rates of lifetime psychedelic use are greater in males than in females. Overall rates of lifetime psychedelic use are roughly the same among the “baby boomers” and younger adults. However, psilocybin was more common among younger adults, while LSD and mescaline or peyote were more common among older adults. Use of psilocybin mushrooms has increased since the 1970s in the US and worldwide, likely due to dissemination of simple home cultivation techniques, instructions on finding wild mushrooms, and information about effects and methods of psilocybin mushroom use ^4^. This was a retrospective cross-sectional study. Self-reports of drug use behaviors could be influenced by memory errors and under-reporting; however, a 14-year longitudinal study reported good consistency over time in the reporting of lifetime LSD use ^5^.

## References

[1] Nichols DE: Hallucinogens. *Pharmacol Ther.* 2004;101(2):131–181 10.1016/j.pharmthera.2003.11.002 14761703

[2] González-Maeso J Weisstaub NV Zhou M: Hallucinogens recruit specific cortical 5-HT(2A) receptor-mediated signaling pathways to affect behavior. *Neuron.* 2007;53(3):439–52 10.1016/j.neuron.2007.01.008 17270739

[3] Hallock RM Dean A Knecht ZA: A survey of hallucinogenic mushroom use, factors related to usage, and perceptions of use among college students. *Drug Alcohol Depend.* 2012;130(1–3):245–8 10.1016/j.drugalcdep.2012.11.010 23265089

[4] Andersson C Kristinsson J Gry J: Occurrence and use of hallucinogenic mushrooms containing psilocybin alkaloids. *Nordic Council of Ministers.* 2009 Reference Source

[5] Johnston LD O’Malley PM: The recanting of earlier reported drug use by young adults. *NIDA Res Monogr.* 1997;167:59–80 9243557

