# Supplementary material for: Over 30 million psychedelic users in the United States
Source: F1000Res. 2013 Mar 28;2:98. [Version 1] doi: 10.12688/f1000research.2-98.v1 (PMC3917651; doi:10.12688/f1000research.2-98.v1)
Supplement: Peer review history

# Peer review: Referee response for version 1

**Published:** 2013-04-17

**Authors:** Dave Nichols

**Affiliations:** 1 Department of Physical chemistry and Molecular Pharmacology, Purdue University, West Lafayette, IN, USA

This report is short and well written.  It reports on lifetime incidence of use of various psychedelics. Without knowing the frequency of use, however, these data do not indicate whether the use of psychedelics is increasing.

I have read this submission. I believe that I have an appropriate level of expertise to confirm that it is of an acceptable scientific standard.

# Peer review: Referee response for version 1

**Published:** 2013-04-03

**Authors:** Wayne Hall

**Affiliations:** 1 University of Queensland, St Lucia, Australia

The paper is concise and well written and I have no major criticisms of the content of the article. The authors have appropriately analysed the best available data on the question that they address namely, what is the estimated life time use of various psychedelic drugs among US adults. They report analyses of a large representative US household survey data on lifetime experience with various psychedelic drugs. My issue is more with what the paper does not say.

What’s missing from the paper is any indication of why the authors’ question is worth posing: 

1. Why do lifetime rates of psychedelic drug use among US adults matter?

2. Why did they only report lifetime use? Lifetime or ever use can overstate rates of use in the population. I would bet that most psychedelic use is very limited e.g. once or twice during late adolescence and early adulthood. This isn't clear from the lifetime data presented. Some data on frequency of use would make it clearer if most psychedelic use is in fact limited and experimental rather than more persistent as occurs with cannabis and MDMA. 

3. Were any data collected on adverse events or experiences? If so, this could be briefly reported.

4. It would be useful to provide a bit more data on the characteristic of psychedelic drug users other than their age and sex. How are they characterised by SES, education, geographic areas of residence and experience with other illicit drugs e.g. cannabis, MDMA and stimulants? 

5. What if anything follows from the findings? Should we be concerned about this use or should we regard psychedelic drug use as being of much lower public health concern than the use of other illicit drugs such as cannabis, amphetamine type stimulants, cocaine and opioids?

I have read this submission. I believe that I have an appropriate level of expertise to confirm that it is of an acceptable scientific standard.
